# Morphology and ultrastructure of retrovirus particles

**DOI:** 10.3934/biophy.2015.3.343

**Published:** 2015-08-18

**Authors:** Wei Zhang, Sheng Cao, Jessica L. Martin, Joachim D. Mueller, Louis M. Mansky

**Affiliations:** 1 Institute for Molecular Virology, University of Minnesota, Minneapolis, MN, USA; 2 Department of Diagnostic and Biological Sciences, School of Dentistry, University of Minnesota, Minneapolis, MN, USA; 3 Wuhan Institute of Virology, Chinese Academy of Science, Wuhan, China; 4 Pharmacology Graduate Program, University of Minnesota, Minneapolis, MN, USA; 5 School of Physics and Astronomy, University of Minnesota, Minneapolis, MN, USA; 6 Characterization Facility, University of Minnesota, Minneapolis, MN, USA; 7 Department of Microbiology, University of Minnesota, Minneapolis, MN, USA

**Keywords:** retrovirus structure, Gag lattice, capsid protein shell, envelop protein complex, cryo-electron microscopy, cryo-electron tomography, sub-tomogram averaging

## Abstract

Retrovirus morphogenesis entails assembly of Gag proteins and the viral genome on the host plasma membrane, acquisition of the viral membrane and envelope proteins through budding, and formation of the core through the maturation process. Although in both immature and mature retroviruses, Gag and capsid proteins are organized as paracrystalline structures, the curvatures of these protein arrays are evidently not uniform within one or among all virus particles. The heterogeneity of retroviruses poses significant challenges to studying the protein contacts within the Gag and capsid lattices. This review focuses on current understanding of the molecular organization of retroviruses derived from the sub-nanometer structures of immature virus particles, helical capsid protein assemblies and soluble envelope protein complexes. These studies provide insight into the molecular elements that maintain the stability, flexibility and infectivity of virus particles. Also reviewed are morphological studies of retrovirus budding, maturation, infection and cell-cell transmission, which inform the structural transformation of the viruses and the cells during infection and viral transmission, and lead to better understanding of the interplay between the functioning viral proteins and the host cell.

## 1. Introduction

Retroviruses are a large group of enveloped, single-stranded RNA viruses that have been associated with a variety of cancers and acquired immune deficiency syndrome (AIDS) [1,2]. The family *Retroviridae* consists of seven genera (*Alpha, Beta, Gamma, Delta, Epsilon, Lentivirus and Spumavirus*) that include many widely studied viruses, including human immunodeficiency virus type 1 (HIV-1, *Lentivirus*), human T-cell leukemia virus type 1 (HTLV-1, *Deltaretrovirus*), Rous sarcoma virus (RSV, *Alpharetrovirus*), murine leukemia virus (MLV, *Gammaretrovirus*) and Mason-Pfizer monkey virus (MPMV, *Betaretrovirus*). In an effort to elucidate the molecular mechanisms responsible for the assembly, maturation and infectivity of these viruses, the structures of retrovirus proteins and particles have been extensively examined using such techniques as nuclear magnetic resonance (NMR), X-ray crystallography, cryo-electron microscopy (cryo-EM) and cryo-electron tomography (cryo-ET).

Retroviruses generally infect the host cell through fusion of the viral and cellular membranes mediated via two mechanisms: pH-independent fusion following interaction with a receptor and co-receptor on the plasma membrane, or low-pH triggered fusion with the endosomal membrane [3,4]. With HIV-1, the viral entry process is orchestrated by the envelope glycoprotein (Env), which is a trimeric heterodimer composed of non-covalently associated gp120 and gp41 subunits derived from a common gp160 precursor [5]. Interaction between gp120 and the cellular receptors induces conformation changes in Env that allow activated gp41 to accomplish its function of membrane fusion, ultimately resulting in delivery of the viral core into the cytoplasm. The viral RNA genome is then reverse transcribed into a double-stranded DNA by reverse transcriptase and subsequently transported to the nucleus, where it is inserted into the host chromosome by the viral integrase protein to form a provirus. The provirus is then replicated along with the host genome and passed on to descendant cells. The provirus is also transcribed into mRNAs by the host cell’s protein synthesis machinery to produce the viral proteins required for assembly of progeny virions. Viral RNA architecture and secondary structure has been shown to contain regulatory structural motifs that play important roles in retroviral replication processes, including the ribosomal frame shift signal and the viral RNA packaging signal [6,7,8].

**Figure 1:**
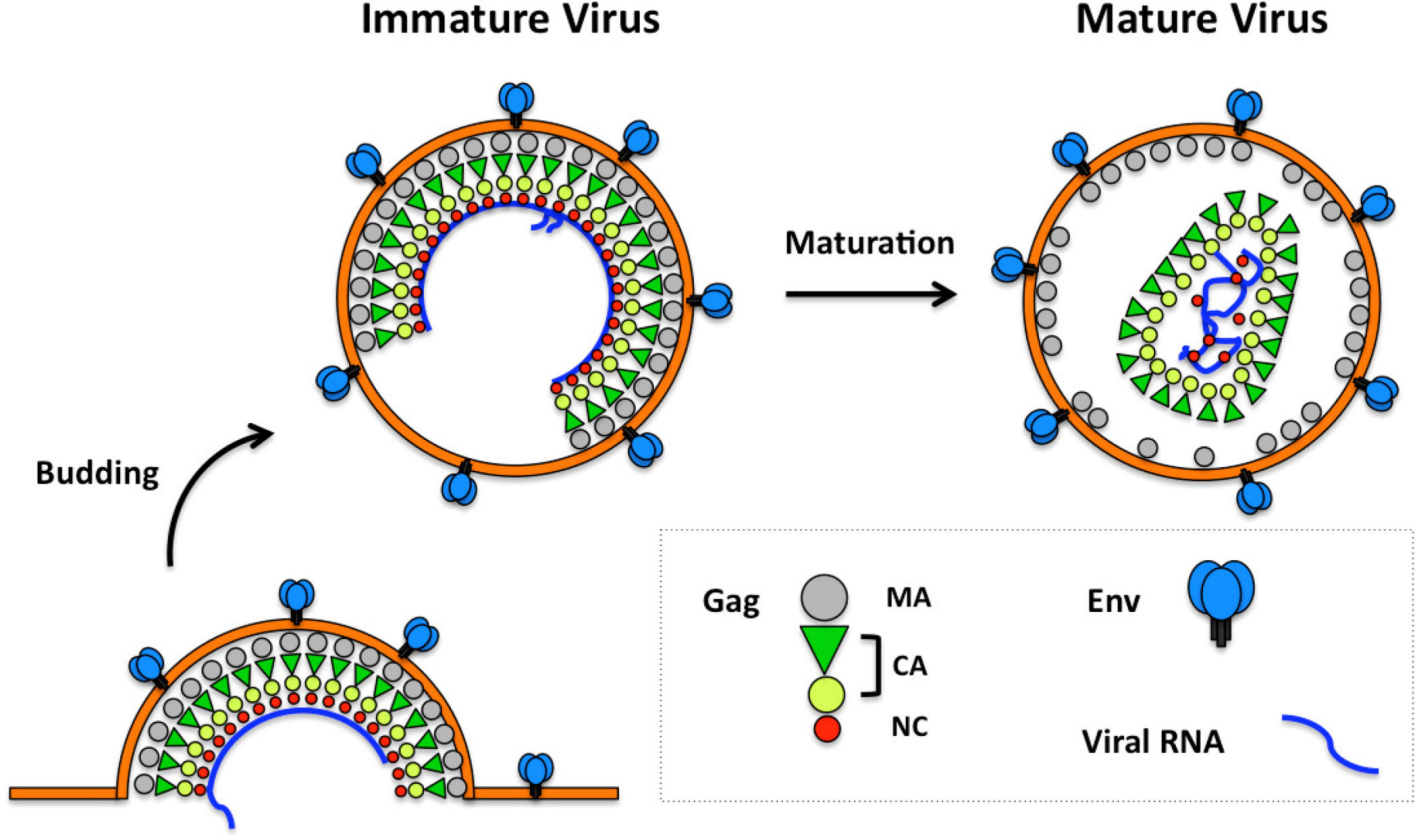
Gag and retrovirus assembly. The cartoon depicts three stages in retrovirus morphogenesis: a partially assembled particle on the host cell’s plasma membrane, an immature particle that is composed of a paracrystalline Gag structure, and a mature virus particle that has a distinct core [13,20]. Gag is a polyprotein including matrix (MA), capsid (CA) and nucleocapsid protein (NC) domains. Env represents the trimeric envelope protein complex. The figure is adapted from two review papers [13,20].

The assembly of retroviruses involves the formation and budding of immature particles on the cell membrane [1,2]. The immature particle is composed mainly of the viral membrane, Env, Gag/Gag-Pol/Gag-Pro-Pol polyproteins [9] and the diploid retroviral genome. Gag proteins organize into an incomplete protein shell that is closely associated with the inner leaflet of the viral membrane (Figure 1). In concert with viral assembly, cleaved trimeric Env complexes at the cell surface are incorporated into the viral membrane through a cell-type dependent process. For example, incorporation of HIV-1 Env into assembled virions is not affected by removal of gp41 cytoplasmic domain in several widely-used laboratory cells lines such as HeLa, COS and 293T, while similar gp41 truncation induces an ~10-fold defect in Env incorporation in the majority of T-cell lines as well as monocyte-derived macrophages [10,11]. Either concomitant or shortly after budding during maturation [12], Gag is cleaved by viral protease into three major proteins: the matrix (MA), capsid (CA) and nucleocapsid (NC), as well as other domains that are virus-specific. Among these, MA remains bound to the inner leaflet of the viral membrane [13,14], while a subset of CA proteins assemble into a capsid, which encapsulates the NC-RNA complexes and viral replication enzymes [15]. Proteolytic cleavage and disassembly of the Gag lattice enables gp41 to assume its fusogenic configuration [16,17]. Activation of gp41 and formation of the viral core with the viral RNA in a proper conformation is required for subsequent membrane fusion and reverse transcription in the succeeding cycle of infection [18,19].

Although the tertiary structures of the MA, CA and NC proteins are conserved among retroviruses [1,2], both immature and mature retroviruses display a heterogeneous morphology, which poses significant challenges for studying the protein organization and protein-protein contacts within these particles. In recent years, the development of *in vitro* assembly systems has provided Gag or CA derived specimens with helical or icosahedral symmetries, which has facilitated image processing and 3D reconstruction. Efficient cryo-EM and cryo-ET data collection procedures and robust computation algorithms have also made high-quality data available in resolving at sub-nanometer resolution the structures of the Gag lattice in immature particles, CA assembly in mature particles, and Env [18,21,22,23]. In addition, cryo-ET and complementary imaging techniques are being used to gain understanding of the retroviral assembly, maturation and infection processes. This review focuses on the current knowledge about retrovirus structures at unique steps in the virus life cycle, as well as relevant technical advancements.

## 2. Retrovirus Morphology—Determining the Size, Shape, Protein Organization and Stoichiometry

Retroviruses are generally spherical enveloped particles with an average diameter ranging between 100 to 200 nm [24–28]. The immature particles display a distinct doughnut shaped morphology in thin section TEM, within which a heavily stained protein density is observed encircling the center of the virus. Cryo-EM images of immature retrovirus particles reveal that the dense layer beneath the immature retrovirus membrane is composed of Gag polyproteins that exhibit a paracrystalline order [24,28,29]. Cryo-ET and sub-tomogram averaging have further demonstrated the arrangement of Gag molecules within purified virions or virus-like particles [30–33] and *in vitro* assemblies [30,31,34]. The MA domain of Gag localizes to the outermost edge of the protein shell, and the NC domain points toward the center of the virion. Both the N-terminal and C-terminal domains of CA proteins (NTD and CTD) form hexagonal arrays with a distance of ~80 Å between the NTD hexamers [31]. In HIV-1, a layer of density pillars, which likely contributes a third layer of hexagonal order beneath the CTD hexamers [33], is interpreted as the putative CA-SP1 boundary that has been predicted to form six-fold symmetric helical bundles [35,36]. The MA segment does not appear to form an extended lattice in the immature virion. Within immature retrovirus particles, the Gag lattices do not fully cover the viral envelope, and large regions beneath the viral membrane lack ordered Gag molecules. It is believed that the curvature of the Gag lattice is not mediated by incorporation of pentameric Gag molecules, but rather by the interspersion of irregularly shaped defects in the Gag lattice [31].

**Figure 2:**
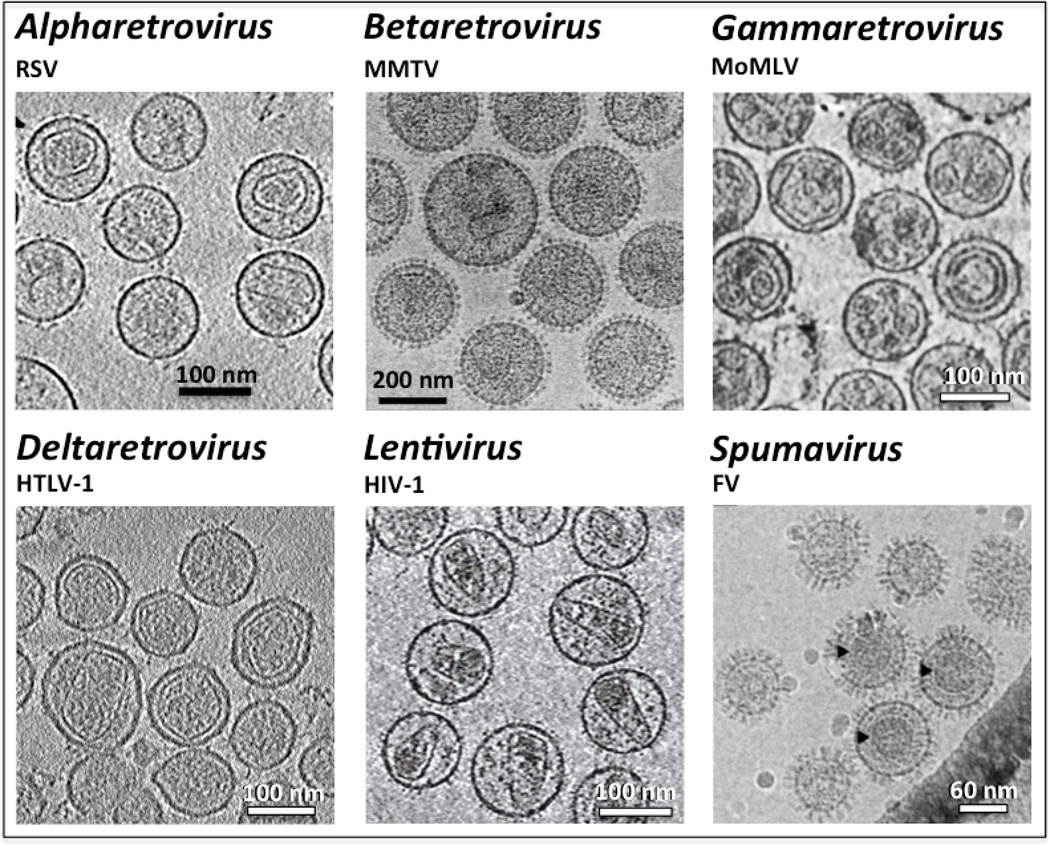
Morphology of mature retrovirus virions. The cryo-EM images and cryo-ET cross sections of the viruses display heterogeneous morphology and size of the viral core in six retrovirus genera. RSV: Rous sarcoma virus [25], reprinted with permission from Elsevier; MMTV: mouse mammary tumor virus [51], amended with permission from American Society for Microbiology; MoMLV: Moloney murine leukemia virus [52]; Copyright (2005) National Academy of Sciences, U.S.A. HTLV-1: human T-cell leukemia virus type 1 [40]; HIV-1: human immunodeficiency virus type 1 [53], reprinted with permission from Elsevier; FV: Foamy virus [54]. The FV Gag protein is not processed into the classical orthoretroviral MA, CA and NC subunits during particle morphogenesis. The black arrowheads show regular Gag assemblies in the wild type virus [54]. Reprinted by permission from the journal.

Within mature viruses, the capsids adopt a variety of shapes and sizes (Figure 2). HIV-1 and *Lentivirus* capsids are primarily cone-shaped [27,28,37,38], while other retroviruses, such as RSV and MLV, display polyhedral or nearly spherical capsids [1,25,27,28,39]. A recent structural analysis of HTLV-1 shows that it has a poorly defined polyhedral capsid, with angular polygon-like regions and at least one curved region in each capsid [40]. As compared to icosahedral viruses, the protein organization within retrovirus capsids is believed to follow the principle of fullerene arrangement, in which a hexagonal capsid lattice containing 12 pentamers form a closed shell. In a nearly spherical retrovirus core the 12 pentamers are distributed randomly [1]. Cylindrical capsids have six pentamers at each end of a tube, and conical capsids have five pentamers at the narrow end and seven at the wide end [41]. From a study of the RSV capsid, it was proposed that a typical RSV capsid, which is a coffin-like fullerene structure, has six five-fold sites at each end distributed in a conventional icosahedral (5+1—i.e. 5 pentamers on the periphery and 1 in the middle) configuration in the cap, while the base has a (6 + 0) configuration if it is flat or a (4 + 2) configuration with the slightly more curved structure [25,39]. The fullerene model provides a mathematical platform that explains the complex and polymorphic retroviral capsid structures.

The average copy number for Gag proteins in retroviruses has been estimated using two techniques: quantitative dark-field scanning transmission electron microscopy (STEM) [42] and fluorescence fluctuation spectroscopy (FFS) [43,44]. With the first method, the average mass of the freeze-dried virus particles is determined and then used to estimate the average Gag copy number in a single immature virus particle [24,45,46,47]. With the second method, the brightness of fluorescently labeled Gag molecules incorporated into the virus particles is measured, enabling direct determination of the number of Gag proteins in each particle [48,49]. It is now known that immature retrovirus virions contain 1500 to 5000 copies of Gag proteins [24,29,37,41,45–48]. Moreover, it was determined using HIV-1 that after Gag cleavage, only a small portion of, the 1500 or so, resulting CA molecules are needed to assemble the HIV-1 core [24]. This result is consistent with the measurement made by hydrogen-deuterium exchange experiment [50]. A calculation made in another study, of RSV, also indicated that the number of assembled CA subunits in a mature virion is less than the number of Gag subunits inferred to be present in its precursor, and that this feature is essential for the infectivity of most retroviruses [25]. Because of ambiguity in the size and shape of retrovirus capsids, an abundance of Gag within retroviruses may be necessary to ensure proper maturation that is linked to the infectivity of retroviruses [21].

Within HIV-1, Env is a trimer composed of the transmembrane glycoprotein gp41 and an envelope glycoprotein gp120 [55]. Cryo-ET and sub-tomogram averaging methods revealed that the HIV-1 gp41/gp120 trimer forms spikes that project away from the viral membrane. The complex has a mushroom-like morphology with a height of ~120 Å and a maximal width of ~150 Å [56,57]. The stalks of the Env spikes in HIV-1 and simian immunodeficiency virus (SIV) display a single-stem configuration [23,58–62]. The Env complex of another retrovirus, Moloney murine leukemia virus, also has a trimeric morphology [63,64,65]. Virions of wild-type HIV-1 and mutant SIV have approximately 14 and 73 spikes per particle, respectively, with some clustering of spikes in HIV-1 [66]. A study of RSV showed that the number of Env spikes is variable (approximately 0 to 118) and correlates with core type; virions with angular cores average 82 spikes, while those with tubular cores average 14 spikes [25]. This suggests the incorporation of retrovirus Env proteins into virus particles is complex and multifaceted. Although, passive incorporation of Env and other cellular proteins may occur, genetic data suggest that, for HIV-1, interactions between the cytoplasmic domain of gp41 and the MA domain of Gag play a major role in Env incorporation in the majority of T-cells and monocyte-derived macrophages [10,11].

## 3. High-Resolution Structures—Examining Protein-Protein Interactions within Retroviruses

The molecular organization of several retroviral structures has been studied at sub-nanometer resolution using cryo-EM single particle reconstruction, cryo-ET followed by sub-tomogram averaging methods and 2D crystallography. By combining these techniques with *in vitro* assembly systems that use purified retroviral structural proteins derived from Gag or CA to imitate retrovirus assembly in a cell-free environment, our current understanding of the structures of immature and mature retrovirus particles was established [21]. Several examples are as follows. An *in vitro* system for MPMV produced tubular structures of a recombinant MPMV CA-NC construct. The structures possessed helical symmetry that reflected Gag assembly in the immature particle [12]. HIV-1 CA proteins were assembled into tubes representing the CA organization in a mature capsid (Figure 5 A) [41,69], while RSV CA was assembled into icosahedral particles that presented CA pentamers not previously visualized in any other *in vitro* assembled system [70,71]. Using cryo-EM helical or icosahedral reconstruction methods, these structures were resolved at sub-nanometer resolutions. In addition, using 2D crystallography the organization of planar full-length CA hexamers was also determined to sub-nanometer resolution [72]. For retrovirus Env, the solution structure of the HIV-1 Env trimer in complex with ligands or in an unliganded state was established to ~6 Å resolution [60,73,74]. Fitting the atomic structures of CA, NC, gp120 and gp41 into the reconstruction maps generated pseudo-atomic models of the Gag lattice, CA lattice and Env spikes. Although these structures were not directly derived from virions, they present a high degree of similarity to the respective authentic virus structures and therefore provide eminently valuable information about the function of these molecules within the retrovirus life cycle.

Recent improvements in cryo-ET and sub-tomogram averaging have dramatically improved the amendable resolution of the immature Gag lattices of MPMV and HIV-1 from 20 Å to 8–9 Å (Table 1) [22,67,75,76]. This was the first time that the structure of a native retrovirus particle was determined at a sub-nanometer resolution. In addition to ensuring homogeneous sample preparation and accuracy of the alignment between the starting model and sub-tomograms, three technical refinements have been critical for achieving high resolution [76]. First, the total electron dose for cryo-ET data collection is significantly reduced. A typical tomographic series uses ~100–200 electrons/Å^2^. The 8.8 Å resolution of the immature HIV-1 particle reconstruction was computed from a tilt series recorded at a dose of ~40 electrons/Å^2^. Second, the defocus level of each image in the tomo-tilt series was accurately determined by setting the view used for focusing very close to the exposure area during data collection. This procedure enabled image collection at very stable defocus through the tilt series. It was suggested that the defocus should be successfully determined, with an accuracy of better than 100 nm. Third, the sub-tomogram averaging employed a huge data set. The highest resolution structure was obtained by averaging over 700,000 asymmetric units. The improvements in cryo-ET and sub-tomogram averaging techniques create numerous opportunities for studying protein arrangement in retrovirus structures.

### 3.1. Immature virus structure

**Figure 3:**
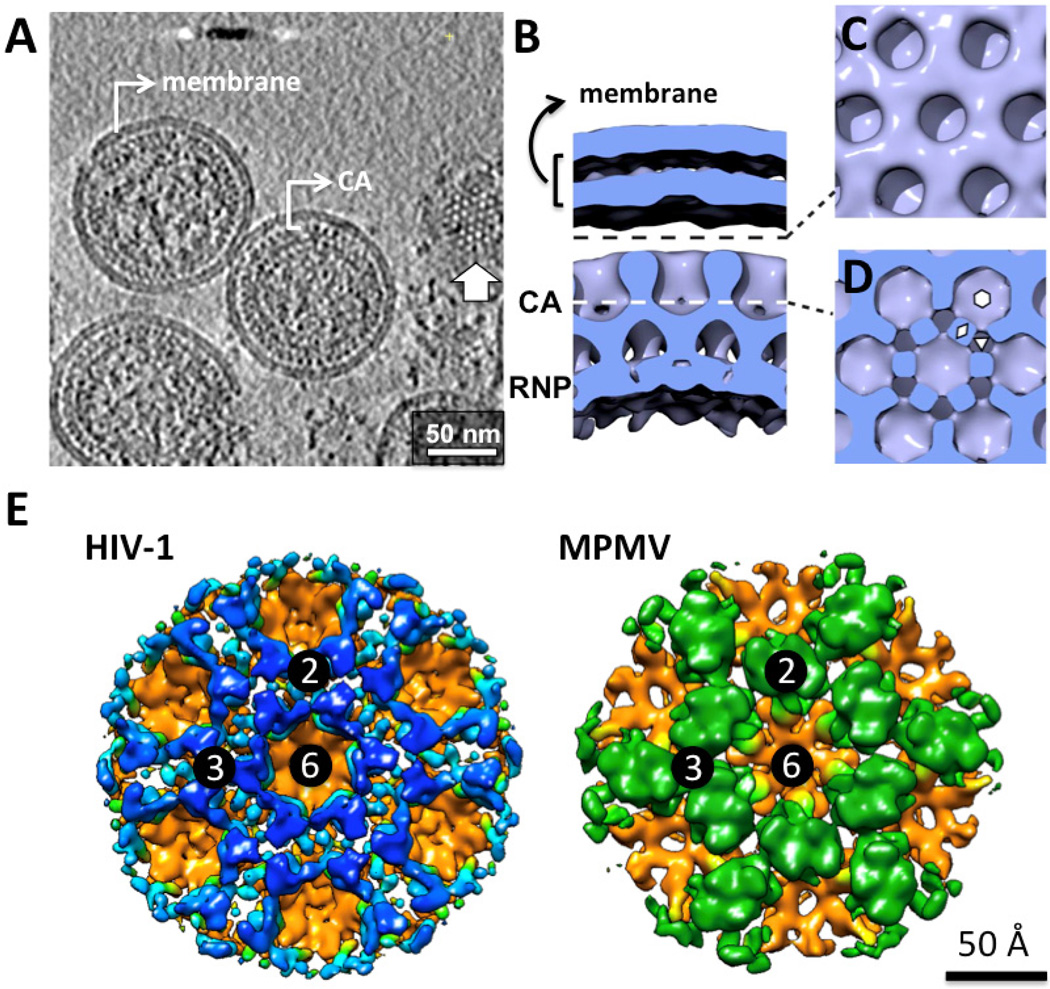
Structure of Gag lattice within immature HIV-1. (A) Computational slice through a Gaussian-filtered tomogram containing immature HIV-1 particles treated with the protease inhibitor amprenavir [67]. The white arrow marks a slice through the CA layer illustrating the hexagonal lattice. Defects of the Gag lattice are evident in these particles. Figure courtesy of John Briggs. (B–D) Surface rendering of the reconstruction of immature HIV-1 particle [31]. Adapted by permission from the journal. (B) A cross section perpendicular to the membrane. RNP represents ribonucleoprotein complex. (C) Surface cut tangential to the membrane at a radius indicated by the black dash line in B, and looking down on the NTDs of the CA lattice. (D) Surface cut through the CTDs of the CA lattice. (E) Surface rendering of the CA organization in the HIV-1 (EMD-2706) and MPMV (EMD-2707) immature particles [67], viewed from the same position shown in C. The CTDs in both viruses are colored in orange, while the NTDs of CA in HIV-1 and MPMV is colored in blue and green respectively. The numbers represent two-fold, three-fold and six-fold symmetry axes. These figures are produced using UCSF Chimera [68].

**Figure 4:**
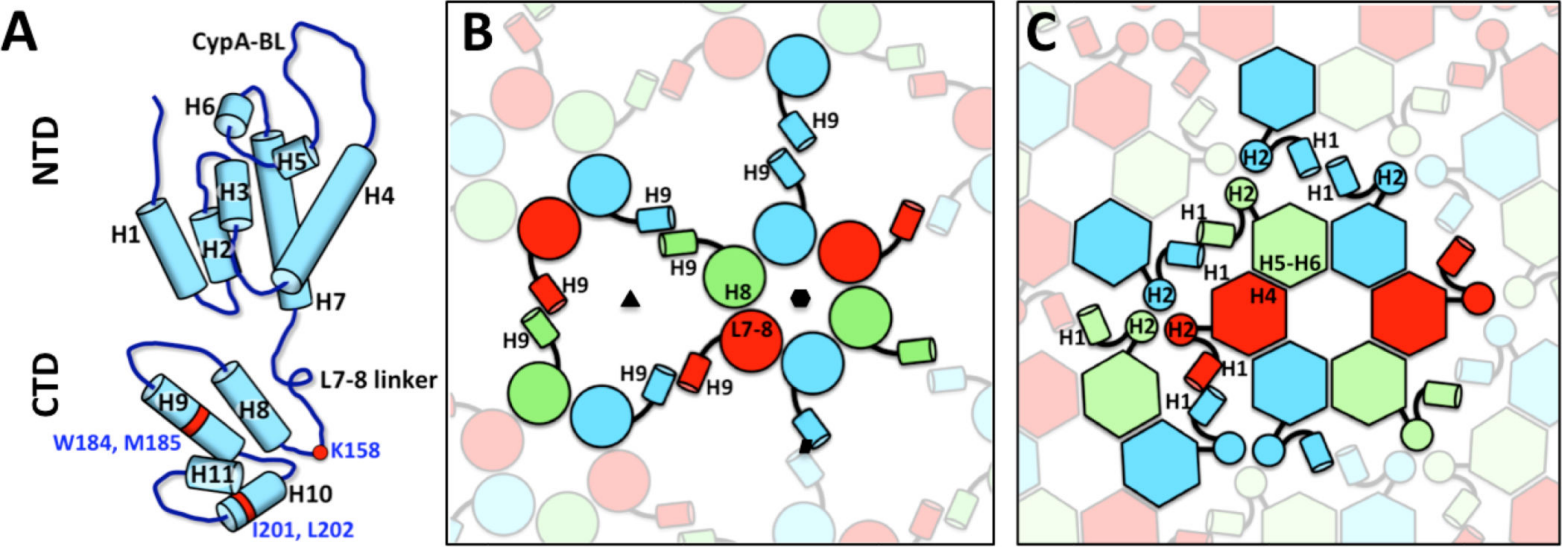
Interactions stabilizing the immature HIV-1 CA lattice. (A) A diagram of CA molecule, indicating sequence of the helices in the NTD and CTD domains. CypA-BL represents Cyclophilin-A binding loop. The diagram was adapted from Figure 2a by Schur et al. [67]. (B) Cartoon that depicts CTD interactions. L7–8 represents the linker region between helix 7 and helix 8. The two-fold, three-fold and six-fold symmetry axes are indicated by a black parallelogram, triangle and hexagon respectively. (C) Cartoon that depicts NTD interactions. The elements that are at the equivalent positions and have the same color code in B represent the same CA molecule.

The most detailed models of immature Gag lattices are based on the structures of *in vitro*-assembled tubular arrays of MPMV CA-NC proteins at 8 Å resolution [12] and the immature HIV-1 capsid within intact virus particles at 8.8 Å resolution (Figure 3 A and E) [67]. The structures of MPMV CA-NC tubes were determined using two methods: helical reconstruction [12] and tomography followed by sub-tomogram averaging [76]. The validity of the modeled MPMV Gag lattice within the tubular structure was confirmed by reconstruction of protease-inhibited immature MPMV virions [67]. Although amino acid conservation between CA proteins from different viral genera is poor, the secondary and tertiary structures are highly conserved [21]. A retrovirus NTD contains seven α-helices and a β-hairpin, while the CTD contains four α-helices and a flexible linker connecting the NTD (Figure 4 A) [72]. The high resolution of these cryo-EM reconstruction maps enabled unambiguous fitting of the atomic structures of the HIV-1 NTD [77] and CTD [78], the NMR structure of the MPMV NTD [79] and the homology model of the MPMV CTD, based on the HIV-1 CTD structure [12,78]. The fitting revealed the positions of the two CA domains and led to generation of pseudo-atomic models of the immature CA lattice in both viruses. These available Gag lattices suggest similar hexameric arrangement of the CTDs in MPMV and HIV-1, but different NTD configurations.

In reconstruction maps of the immature Gag lattices from MPMV and HIV-1, the CTDs are organized as hexamers that interface with each other through a homodimeric interaction. Fitting the HIV-1 CTD into the HIV-1 immature Gag lattice revealed two critical molecular interfaces (Figure 4 B) [67]. The first locates in the contact area between adjacent CTD monomers within the hexamer and involves residues near helix 8 and the linker between helices 7 and 8 (Figure 4A–B). This location corresponds to part of the major homology region (MHR) [88,89] and is consistent with a role in Gag lattice assembly and hexamer stabilization. The second interface involves the packing of CA helix 9, including two critical hydrophobic residues (W184 and M185) [90] that mediate the homodimeric interaction between pairs of neighboring hexamers (Figure 4B). These molecular contacts are similar to those in the *in vitro*-assembled tubular arrays of HIV-1 [80]. In the immature HIV-1 particle map, densities at a lower radius of the CTD domains were observed having a tube-like structure of six-fold symmetry, which is consistent with previous models suggesting this region assembles a six-helix bundle of SP1 domains [33,67].

The molecular organizations of NTDs within immature MPMV and HIV-1 particles are completely different from one another [12,67]. In MPMV, NTD dimers locate above the two-fold positions between neighboring CTD hexamers. Through the dimeric interactions, and trimeric interactions at the three-fold positions, the MPMV NTD interconnects CTD hexamers. There is no contact between neighboring MPMV NTD dimers around the local six-fold axis. By contrast, NTDs within immature HIV-1 particles are organized into hexamers that make up an extensive network of intra- and inter-hexamer interactions. The molecular interactions between NTDs within HIV-1 include contacts around local six-fold axes that involve the C-terminal end of helix 4 in one monomer and the loop between helices 5 and 6 in another, three-fold bundle interactions contributed by helix 2 at the three-fold axis, and a homodimeric interface formed between two helix 1 segments from two adjacent hexamers (Figure 4C). These findings suggest that MPMV and HIV-1 CA proteins adopt different quaternary arrangements within immature virus particles, even though the tertiary structures of the two domains within CA are conserved. No substantial NTD and CTD interfaces were detected in either MPMV or HIV-1 Gag lattices.

The determined molecular contacts within immature HIV-1 virus structures are consistent with the functional regions at the surface of CA previously identified in mutagenesis studies as critical for Gag assembly or capsid formation. Mutations in the CTD and helices 4 and 5 of the NTD interfere with Gag-Gag interactions and particle assembly, while mutations in the N-terminal segments of the NTD (helices 1 and 2) impact capsid formation and viral infectivity. For example, mutation at residue K158 in the MHR of HIV-1 causes a Gag assembly defect that abolishes infectivity. Crystal studies showed helix 9 to be critical for formation of CTD dimers [91], and mutation of residues at the dimer interface (W184A and M185A in the CA sequence) measurably diminished immature particle production and reduced intermolecular Gag-Gag interaction *in vitro* [89,90]. Within the NTD region, a series of mutations in helices 4 to 6 impacted Gag assembly and diminished both viral particle production and capsid formation. Mutations in helices 1 and 2 did not block capsid formation but greatly reduced viral infectivity [89]. A comprehensive list of viral assembly phenotypes of the CA mutants is described in von Schwedler et al. [89]. The cryo-EM reconstructions depicting the spatial arrangement of these residues within immature HIV-1 particles [12,67] can help enhance understanding of the consequences of point mutations and facilitate design of mutants useful for studying the synergetic relations of the functional elements in CA proteins.

Although no organized MA or NC domains have been observed in high-resolution immature virus structures [12,67], these two domains play significant roles in the assembly and formation of the Gag lattice. Studies of HIV-1 Gag trafficking and assembly have shown that interactions between Gag molecules in the cytoplasm trigger the exposure of a hydrophobic myristoyl acid moiety in MA that targets Gag to the inner-leaflet of the plasma membrane through interaction with lipid phosphatidylinositol 4,5-bisphosphate (PI(4,5)P2) [92,93]. The membrane interaction allows more than a thousand of multi-domain and flexible Gag molecules to concentrate, align and form extensive paracrystalline molecular contacts. Gag myristoylation and interaction with the plasma membrane also prevents HIV-1 Gag from forming unproductive oligomers in the cytoplasm [94,95]. In addition, through interaction with the Gag NC domain, the viral genome plays a structural role in Gag-Gag interactions for HIV-1 [96,97]. Immature Gag lattice has been observed in particles that are in the budding process [29]. Formation of lattice is probably the most efficient way for a viral particle to assemble enough copies of Gag to achieve a completely enclosed viral capsid after the maturation process. In a recently study, ordered MA lattice structures were observed near the flat membrane area of a large membrane-enclosed multi-core structure in supernatants of HIV-1 infected cells [86], opening up a new avenue in structural investigation on the ordered arrangement of membrane-bound MA and its role in formation of immature lattice and Env incorporation.

### 3.2. Mature virus capsid structure

**Figure 5:**
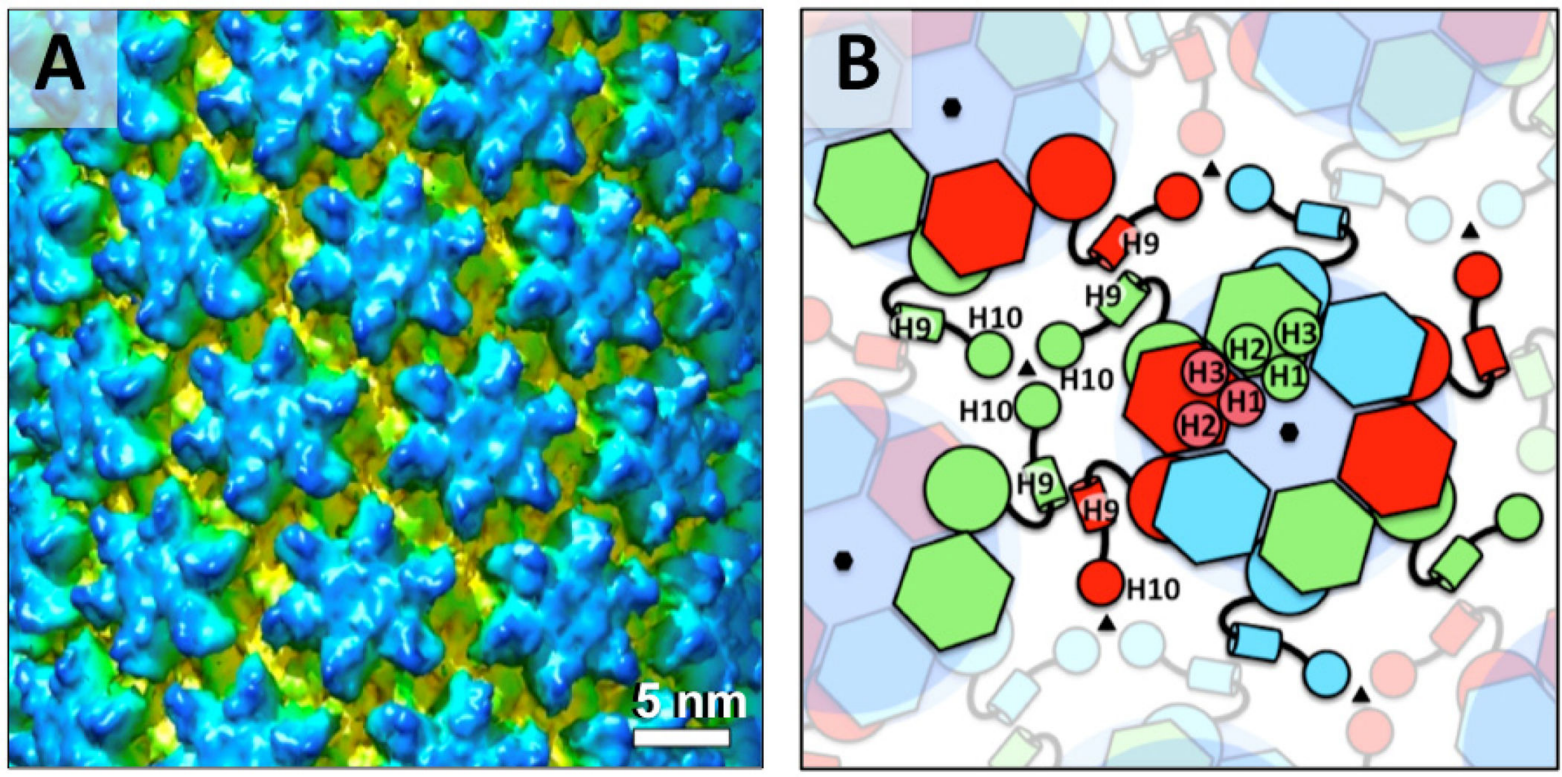
Mature HIV-1 capsid structure. (A) Cryo-EM reconstruction of recombinant A92E CA tubular assembly with (−12, 11) symmetry (EMD 5582, [69]). The hexamer rings of NTDs are shown in blue. The CTDs are shown in yellow. Figure courtesy of Peijun Zhang. (B) A schematic diagram showing interactions between the CA proteins in mature HIV-1 capsid. The hexagons represent NTD and the circles represent CTD. Connected elements in the same color are from the same CA molecule. H1, H2, H3, H9 and H10 represent helices 1, 2, 3, 9 and 10 respectively.

Retroviral capsids are closed protein shells that adopt heterogeneous morphologies such as tubular, conical, nearly spherical or polyhedral (Figure 2). Even within the same virus genus, the capsids are polymorphic and can vary in both size and shape, suggesting substantial flexibility is required when the building blocks of the capsid interconnect with one another to form a protein shell. This variability is in stark contrast to the icosahedral viruses, within which the viral capsid proteins generate spherical protein shells with a relatively uniform curvature. To achieve this flexible and closed capsid architecture, retroviruses utilize CA, a protein composed of two domains connected by a flexible linker. Our current knowledge about the molecular organization of retrovirus capsids is based on studies of HIV-1 and RSV, which contain capsids composed mainly of a lattice of hexagonal or pentameric rings of CA proteins. The hexameric or pentameric CA NTDs form the stable body of the ring, while the CTDs comprise the floor and give flexibility to the capsid shell. The NTDs mainly form intra-hexamer, intra-pentamer and NTD-CTD contacts, while the CTDs additionally sustain inter-hexamer or hexamer-pentamer contacts (Figure 5B). Molecular contacts determined by fitting the atomic structures of CA proteins into reconstruction maps provide valuable information about capsid organization and the flexibility that enables a continuously curved surface to be generated.

The organization of NTD hexamers in HIV-1 and RSV was found to be rigid, stable and consistent in several different assembled forms, including helical structures [41,69], the icosahedral structures of RSV [70] and 2D and 3D CA crystals [78,98,99]. The NTDs are packed to form a flat ring about 40 Å thick and 90 Å in diameter, with a 20-Å channel that passes through the center [99]. Within the crystal structure of a HIV-1 CA hexamer, the first three helices of each CA subunit combine to form an 18-helix barrel at the center of the hexamer [78]. This is consistent with the observed NTD-NTD interface in the RSV icosahedral capsid, where helices 1 and 2 of one NTD are in close proximity to helices 1 and 3 of their neighbor [70]. It is believed that the major molecular contacts between HIV-1 NTDs are through hydrophilic interactions within a hydrogen-bonding network [78]. A similar polar environment created by bridging water molecules was also observed in the hexameric X-ray structure of the isolated NTD from the MLV CA [99].

Within the CA lattice of a retrovirus capsid, the CTDs form inter-hexamer connections. Cryo-EM reconstruction of helical HIV-1 structures [69,100] revealed that neighboring CTDs interact at local quasi-two-fold and three-fold axes. The hydrophobic residues W184 and M185 in helix 9 contribute CTD contacts at the local two-fold positions. At the three-fold interfaces, a hydrophobic patch composed of I201, L202, A204 and L205 on helix 10 (Figure 4A) is critical for stabilizing the contacting interface, which is further stabilized by nearby oppositely charged polar residues (K203 and E213). When the residues of the hydrophobic patch were replaced with different hydrophobic residues, capsid stability and infectivity were retained, but infectivity was lost when the polar residues were used for the mutation at the hydrophobic patch. On the other hand, a mutation that induced disulphide cross-linkage of CA proteins significantly increased the efficiency of capsid assembly both *in vitro* and in mature virions. In addition, it has also been shown that a host anti-viral protein, tripartite motif protein isoform 5 alpha (TRIM5α), can bind to the HIV-1 capsid and weaken the CTD trimer interface [101,102], which causes premature capsid disassembly and inhibits HIV-1 infection. The trimeric interaction of CTDs thus plays a crucial role in capsid stability and the infectivity of the virions. It is worth noting that over-stabilizing the capsid may also result in low virus infectivity. For example, the antiviral compound PF-3450074 (PF74) has been found to bind at the NTD-CTD interface of HIV-1 CA proteins. Such interaction leads to inefficient uncoating and reverse transcription. A recent study of X-ray crystal structure of native full-length HIV-1 CA proteins in complex with PF74 revealed the significant role that structured water and PF74 molecules play in capsid stabilization [103].

Fitting both CA hexamers and pentamers into tomographic reconstruction maps led to an understanding of how the curvature of a mature virus capsid is generated and the key factors that stabilize the core structure. A sheet of CA lattice can bend through two mechanisms: in-plane tilt of hexamers in smoothly curved regions and incorporation of pentamers at sharp turning points. In helical HIV-1 structures, interactions between dimers of helices 9 were found to be variable and to exhibit a crossing angle of 36°, 44° and 54° [69]. This flexibility is believed to introduce the curvature of the CA lattice into helical tubes. In RSV, it was found that a helix-capping, hydrogen-bonding interaction between helices 4 and 8 is involved in pivotal movement between the NTD and CTD, suggesting a flexible interface can be formed between the two domains of CA proteins [98]. A similar observation was also made for HIV-1 hexagonal CA sheets [70,78,98,100]. Additional understanding of the structure of CA pentamers has come from molecular modeling using the tomographic reconstruction of HIV-1 capsids [69,100] and the icosahedral reconstruction of RSV CAs [70]. The fitting generated a possible pentameric model of CA that aids in understanding how a fullerene core is generated.

### 3.3. Env complex and its dynamic changes when interacting with antibody and receptors

One important mechanism for retrovirus entry into cells is receptor-mediated membrane fusion. With HIV-1, the Env apex component gp120 first interacts with its cellular receptor, CD4 [104], which is followed by association of a co-receptor, the chemokine receptor CCR5 or CXCR4 [105,106]. These interactions lead to exposure of the fusion domain of gp41 and its ultimate insertion into the cell membrane while the C-terminal helices of gp41 continue to associate with the viral membrane. Subsequent membrane fusion occurs simultaneously with further rearrangement of gp41 that involves folding-back its N-terminal helices to join the C-terminal, membrane-associated helices forming a six-helix bundle [107–110]. A number of broadly neutralizing monoclonal antibodies (bn-MAbs) and peptides are able to block membrane fusion through interaction with gp120, and the N- or C-terminal helices of gp41. Structural studies of HIV-1 Env have focused on understanding the conformational changes in gp120 during interaction with its receptor/co-receptor, the structure of gp41 in its pre- and post-fusion states, as well as the neutralization mechanism manifested by a broad panel of MAbs.

Monomeric HIV-1 gp120 cores, which contain deletions of the V1, V2 and V3 variable loops and of the N- and C-termini compared with full-length gp120, in the unliganded state or in complex with various neutralizing /non-neutralizing antibodies and with reagents that target the CD4 binding site have been extensively studied using X-ray crystallography [23,111–120]. The post-fusion crystal structures of the gp41 core, the α-helical components of gp41 ectodomain, have also been well described [107–110]. These studies revealed the atomic structures of the gp120 and gp41 cores in specific functional states, as well as the molecular interfaces between gp120 and its binding ligands. However, no crystal structure of trimeric Env, in either its native or antibody-bound state, has yet been successfully studied. Cryo-ET reconstructions of the entire HIV-1 virion and closely related SIV virions at ~20 Å resolution [56–62,66,73,74,75,121–124] depict the structural features of Env in its native trimeric form and in complex with various ligands. In 2002, SOSIP, a stable homogeneous gp140 trimer with favorable antigenic properties, was created by introducing an intermolecular disulfide bridge between gp120 and gp41 and an additional mutation into gp41 [125]. This construct led to successful reconstruction of Env trimers and their complexes with several ligands at sub-nanometer resolution [60,73,74,75]. The structural studies that made use of X-ray crystallography, cryo-ET and cryo-EM reconstruction provided insights into the structure of Env and the dynamic conformational changes it undergoes during viral entry and neutralization [23,118].

**Figure 6:**
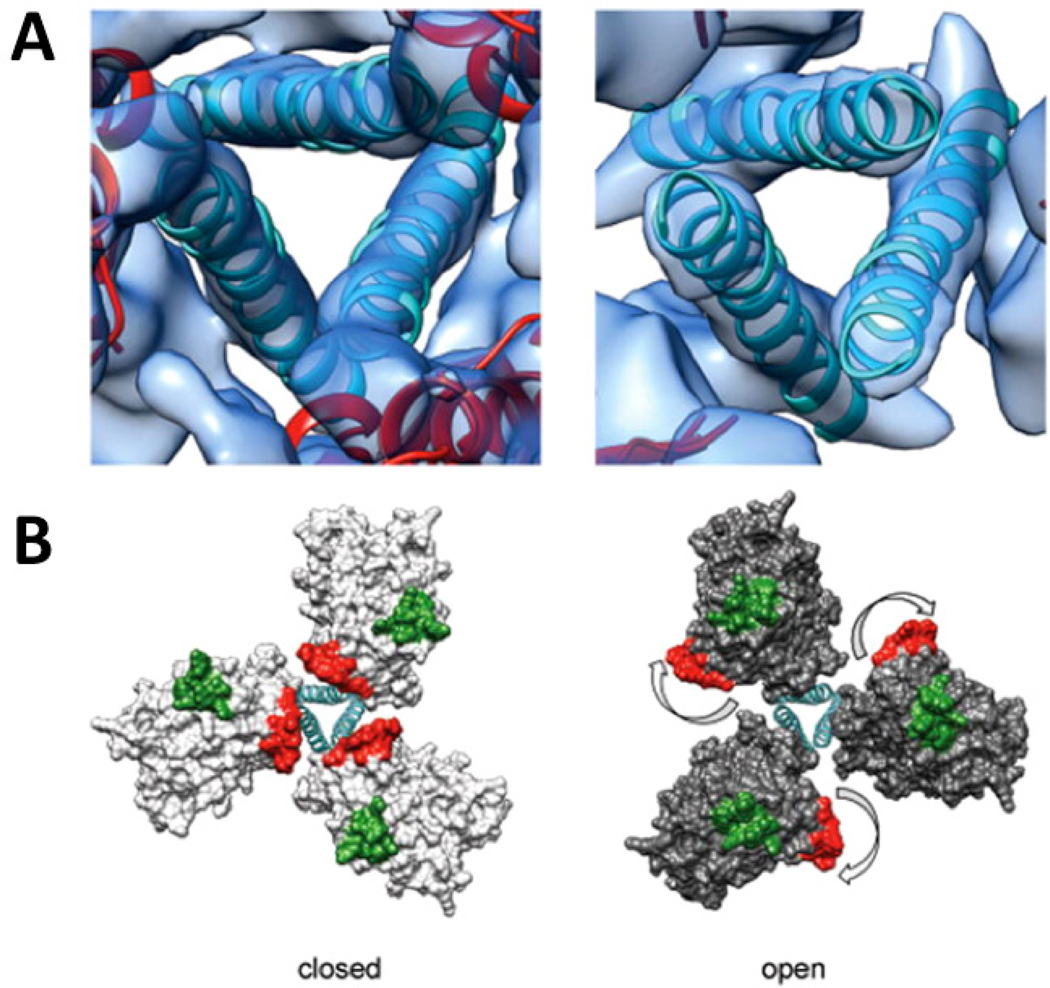
Comparison of structures of trimeric Env in the closed, pre-fusion and open, activated conformations. (A) Zoomed-in top views of the closed (left) and open (right) quaternary states derived from structures of trimeric HIV-1 Env in complex with VRC03 and 17b antibodies, respectively. The ribbons representing the central helices are in an identical position in both panels indicating that the location of the central density is approximately the same in closed and open quaternary conformations. B) Molecular models for the two conformations in (A) show how Env activation results in major rearrangements of gp120 location relative to the central gp41 stalk. The outward rotation of each gp120 protomer repositions the V1V2 loop (base of loop shown in red) from the center to the periphery and alters its position relative to the location of the V3 loop (base of loop shown in green). Reprinted by permission from Macmillan Publishers Ltd: Nat. Struct. Mol. Biol. [73], copyright (2013).

The studies summarized above suggested that when the Env trimer is in an unliganded state, it adopts a closed conformation in which the variable loop regions V1/V2 of gp120 are located close to the apex of the Env spike, while gp41 and its fusion peptide are buried [56,57,66]. Binding of gp120 to its cellular receptor, CD4 [56,62], CD4 binding site (CD4bs) MAbs or CD4 plus CD4-induced (CD4i) MAbs, such as 17b [60] or the llama antibody fragment m36 [59], induces Env to assume an open configuration, in which the V1V2 loop rotates and moves to the periphery of the Env trimer, enabling gp41 to interact with the cell membrane (Figure 6) [58,60,61,122]. When the Env trimer is bound to a CD4bs bn-MAb, such as VRC01, VRC02, VRC03 [60,73] or PGV04 [74], it is locked in its closed state, which blocks co-receptor binding, gp41 activation and subsequent steps toward membrane fusion.

The cryo-EM structure of the gp140-PGV04 complex at 5.8 Å resolution [74] shows the spatial arrangement of the Env components in the closed configuration. The gp120 subunits are assembled into a trimer through intra- and inter-protomer interactions at the apex of the trimer density composed of the variable loops (V1, V2 and V3). Each gp41 protomer, situated at the base of gp140, features two prominent helices, presumably contributed by the heptad repeats HR1 and HR2. HR1 forms a characteristic three-helix bundle in the trimer core, while HR2 is situated around the periphery of the gp140 base. In another study, the cryo-EM structure of the gp140-VRC03 complex at 6 Å resolution suggested the positions of the three central gp41 helices are unchanged in the closed and open conformations, whereas the location and orientation of gp120 differ significantly (Figure 6). This suggests the central gp41 helix-bundle functions as an anchor around which the gp120 subunits pivot outwards during the transition from the closed to open states. A more complete list of antibodies, their binding locations on Env, how they change the morphology of Env trimers and why they function to block viral entry can be found in a pair of review papers [23,126].

## 4. Retrovirus Structures in a Cellular Context—Studying Budding, Maturation, Fusion and Cell-Cell Transmission

One of the greatest obstacles to obtaining structural information about retroviruses inside host cells at nanometer resolution is the comparatively large size of the host cells [127]. For example, CD4+ T cells, which are natural target cells for HIV-1 infection, range from 6 to 10 µm in diameter. This dimension is much larger than the size limit of a specimen (up to 0.5–1 µm) accessible for TEM imaging. Several approaches have been undertaken to overcome this barrier. One utilizes cells that are amendable to producing retrovirus particles, can grow while adhering to the carbon surface of an EM grid and have very thin peripheral areas (e.g. <500 nm) suitable for TEM imaging and cryo-ET reconstruction. Examples of such cells include human glioblastoma cell lines (U-87 MG and U-373 MG cells), HeLa cells at the mid-point between two mitosis phases [128,129], and human umbilical vein endothelial cells (HUVECs). Another approach is to prepare thin-sections of retrovirus infected cells. Electron tomography and 3D reconstruction are then performed using fixed and stained thin-section materials at room temperature [130,131]. A third approach to revealing 3D structural information within or between cells is to employ focused ion beam scanning electron microscopy (FIB-SEM) [132,133,134]. These methods, in conjunction with fluorescence light microscopy [135,136,137], provide valuable structural information about retrovirus budding, maturation, infection and cell-cell transmission.

The budding profile of native HIV-1 particles has been examined in HUVECs using cryo-ET [138]. The budding particles present a half-dome shape with protein densities resembling the Gag lattice defined in immature HIV-1 particles. Some budding particles appear closer to egress and are linked to the cell surface by a narrow membrane neck. In some cases, the same Gag lattice appears on the surface of the cells as islands or flat sheets loosely arranged around a center point. Another study demonstrated HIV-1-like particle assembly and release in human glioblastoma cells [139]. The budding sites were found to be at the plasma membrane on lamellipodia-like structures rich in actin filaments. Cryo-ET sub-tomogram averaging revealed that the Gag organization at individual budding sites is the same as that determined in immature retrovirus particles. In both studies, mature HIV-1 particles could only be found outside the cell, suggesting protease cleavage and formation of the viral capsid of most viruses is completed after completion of virus budding, while the HIV-1 protease is believed to be activated during Gag assembly [2]. The precise mechanism of membrane fission during virus budding is not well defined. It is believed that endosomal sorting complexes required for transport (ESCRT) machinery [140,141,142] recruited directly by the p6 domain in HIV-1 polymerizes into a dome-like structure that promotes membrane fission at the end of virus budding [2,143]. However, no dome-like structure attributable to ESCRT and associated with budding HIV-1 particles has yet been identified.

Retrovirus maturation [2,18] involves two major successive steps: regulated proteolysis and assembly of the mature viral capsid. The proteolytic cleavage is a process whereby activated viral protease dimers cleave the Gag polyproteins into separate domains that include MA, CA, NC and various small peptides. Cryo-ET studies using different HIV-1 cleavage site mutants [138,144,145,146] and HIV-1 maturation inhibitors [32,147] have revealed the intermediate structures between the cleavage events. These studies suggest that for HIV-1, cleavage upstream and downstream of CA-SP1 is essential for immature lattice disassembly, and cleavage within CA-SP1 is required for subsequent formation of the mature lattice [32,144]. The structural mechanism for assembly of the CA capsid is not fully understood. In the case of HIV-1, although both Gag in the immature lattice and CA in the mature lattice exhibit hexagonal organization, the molecular details of these assemblies are quite different. Within the immature particle lattice, the organization of CA is constrained by membrane-bound MA at its N-terminus and RNA-bound NC at its C-terminus, and the hexamer-hexamer interaction among CA proteins is mainly mediated by the NTD. In the mature particle, by contrast, CA is liberated from MA and NC, and the inter-hexamer interaction is accomplished by CTD. Based on the latest sub-nanometer resolution structures of HIV-1 immature [67] and mature lattices [69], hydrophobic dimeric interactions involving helix 9 in CA is the only molecular interface shared by the two structures. It is not clear whether the immature Gag lattice needs to be fully disassembled into monomers, dimers or hexamers before the CA lattice can be established [32,67]. There have been a number of cryo-ET studies into how the CA sheet grows and builds [32,53,138,148,149,150]. One recent work [138] indicated that assembly of the HIV-1 core begins with a small CA lattice sheet that associates with the condensed NC-RNA complex, after which it grows along one side of the conical core toward the tip and then curves around to form a closed shell. A non-diffusional phase transition model was also proposed recently in which the detaching layer of the cleaved CA lattice is gradually converted into a roll that ultimately forms the surface of the mature conical core [86].

Pre-fusion interaction between SIV/HIV-1 and CD4+ T cells was studied using electron tomography with thin-sectioned samples [151]. Within both viruses, a cluster of five to seven rod-shaped structures, termed as “entry claws,” were observed at the virion-cell interface. These distinctive structural features, attributable to Env glycoproteins, were missing when the viruses were incubated with T lymphocytes in the presence of ligands that blocked interaction between the virus and its receptor or co-receptor. Similar structures were also observed in a cryo-ET study of avian sarcoma/leukosis virus (ASLV), an alpharetrovirus, in complex with liposomes in the presence of the virion’s receptor at neutral pH [152]. ASLV requires both receptor binding and low pH for membrane fusion. The virions were observed to tightly bind liposomes through spike-like structures at the junction. The number of spikes per junction varied from 4 to 47, roughly in proportion to the size of the contact area. In these two studies, remarkably similar structures that formed between the retrovirus and a target membrane receptor mediated membrane interaction. The glycoproteins in the virion-cell or virion-liposome complexes were found to be more concentrated at the virion-membrane interface than elsewhere in the viral envelope, suggesting rearrangement of Env positions during formation of the membrane contacts. In addition, multiple copies of Env spikes were present at the virion-membrane junction. It is not clear at this point whether a subset of Env spikes or all of them are directly involved in merging the apposed membranes, and leading to the formation of the post-fusion six-helix bundles of their fusion proteins.

**Figure 7:**
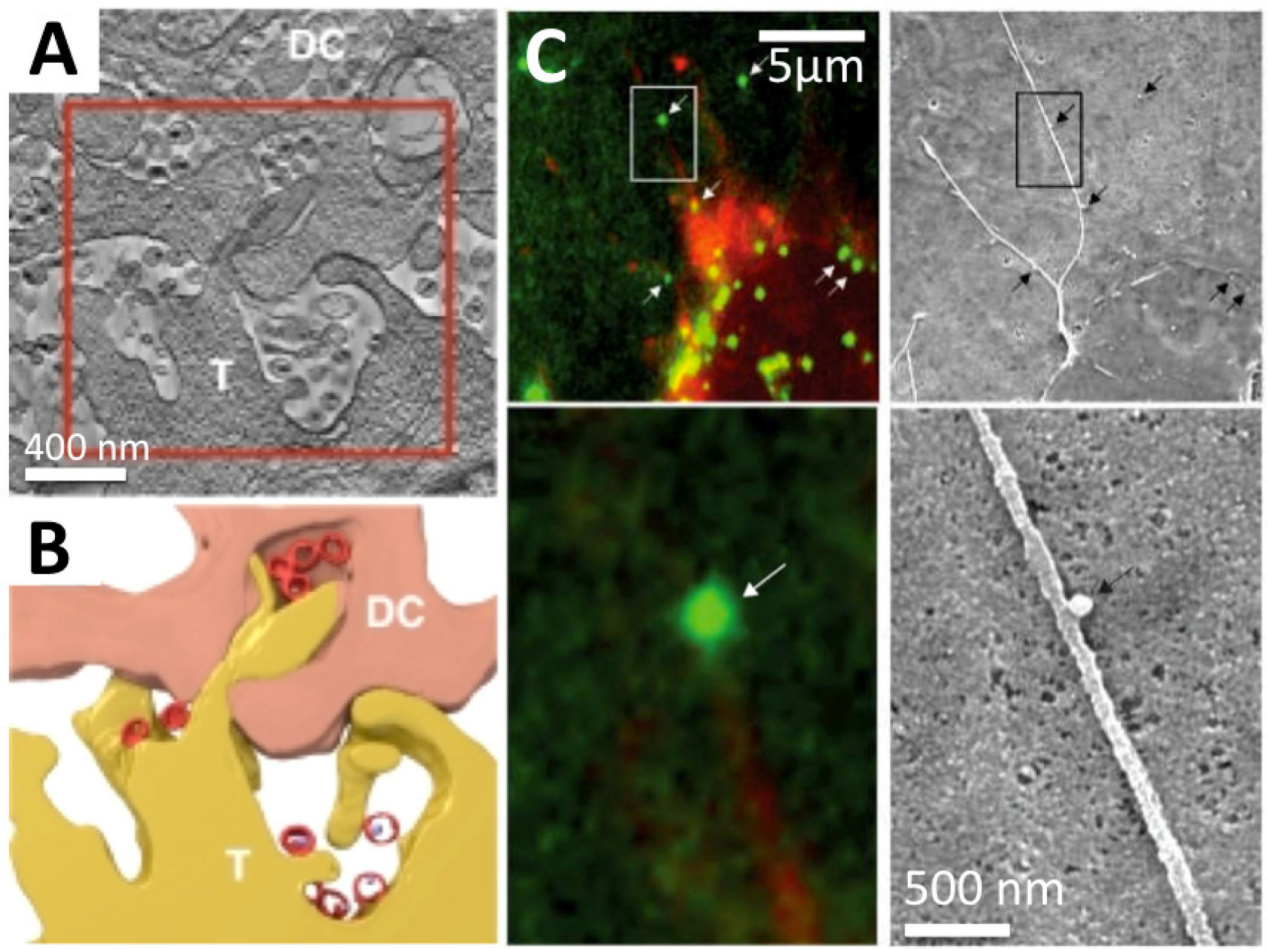
Virus-cell interactions and cell-cell transmission [153,154]. (A–B) A contact region between HIV-1-pulsed dendritic cells (DC) and CD4+ T cells (T) illustrating HIV-1 distribution within the virological synapses [153]. Reprinted by permission from the journal. (A) A ~200 nm thick tomographic slice obtained from a FIB-SEM reconstruction map. (B) Segmentation of the region boxed in A to indicate membrane contact and virus (red) location. (C) MLV moves along the outer surface of filopodial bridges toward target cells [154]. Fluorescently labeled viral particles (green, MLV Gag-CFP; white arrows) moving along filopodial bridges (red, mCAT1-YFP) correlated to single approximately 100 nm particles observed by SEM (black arrows). The boxed areas in the upper panels are magnified in the lower panels. Reprinted by permission from Macmillan Publishers Ltd: Nature Cell Biology [154], copyright (2007).

It has been suggested that cell-to-cell transmission is an effective mechanism by which retroviruses evade the host immune system and infect other cells. The donor cells pass nascent viruses by forming virus-induced cellular structures at cell-cell junctions, such as viral synapses at the junction between two T cells or between a dendritic cell and a T cell, and at cellular conduits or filopodial bridges and the extracellular matrix [155]. Using correlative fluorescence microscopy, electron tomography and FIB-SEM, the cell-cell junctions induced by retrovirus infection have been studied in 3D. The synaptic cleft between a HTLV-1-infected lymphocyte and an autologous target lymphocyte was found to be surrounded by tightly apposed membranes [130]. In contrast, the T cell viral synapse induced by HIV-1 is relatively permeable and accessible to inhibitors [131]. And at the cell junction between an HIV-1-infected dendritic cell and a recipient CD4+ T cell, the dendritic cell was observed to encase the T cell in membranous sheets creating extensive membrane contacts between the two cells [126,132,153]. The HIV-1 virions retained within the compartments on the surface of the dendritic cell could then establish infection through the filopodial structure originating from the T cell (Figure 7 A–B). In addition, in several cell types, MLV particles were observed to establish new infections by moving from infected to target cells along thin, elongated filopodia (Figure 7 C) [154]. These structural studies indicate that the mode of cell-to-cell transmission employed by retroviruses is specific to the types of virus, producer cells and susceptible cells.

## 5. Conclusion and Future Prospect

We look forward to future EM-related studies on the architecture of retroviruses. The individual domains of retroviral CA proteins share remarkably similar tertiary structures. However, recent reports on the sub-nanometer structures of immature Gag lattices from MPMV and HIV-1 suggest that knowledge about Gag-Gag assembly in one virus cannot be directly generalized to equivalent organizations in other retroviruses (Figure 3 E) [67], even though the two Gag lattice structures appear similar at ~27 Å resolution [34]. It is also evident that CA proteins form variously shaped capsids in different mature retrovirus particles. Therefore comparative studies at sub-nanometer resolution about the Gag and CA lattices in different retrovirus genera would greatly enhance our understanding of the architecture of retroviruses. In addition, the structural mechanism of the interactions between retrovirus Env proteins and Gag protein lattice in an immature particle remains elusive. Many studies have suggested that Env incorporation is a cell-type dependent process that might require specific interactions between the cytoplasmic domain of gp41 with MA [10,156,157]. Such interactions have not been revealed by previous cryo-ET studies. Furthermore, several important protein complexes and structure transition intermediates are yet to be resolved. For example, the structure of ESCRT complexes that promote membrane scission after budding and the protein arrangement of the viral fusion proteins in the middle of membrane fusion have not been determined. The application of direct electron detection technologies and enhancement of sample preparation, cryo-EM/cryo-ET data collection and computation schemes [22] would increase our ability to examine at the sub-nanometer level or better the structures of additional retrovirus immature/mature lattices and intermediate structures that have significant implication in the dynamic cellular processes during the virus life cycle.

## Figures and Tables

**Table 1:** Deposited retrovirus reconstruction maps at sub-nanometer resolution.

	Virus	Map Name	EMDB Entry	Rec. Method	Resolution (Å)	Model PDB Entry	Ref.
	
Gag Lattice	MPMV	CANC tube^1^	2089	Helical 7	8.0		[12]
		CANC dimer	2090	Helical	7.0	4ard, 4arg	[12]
		CANC tube	2487	Sub-tomogram 8	8.5		[76]
		CANC dimer	2488	Sub-tomogram	8.3		[76]
		Intact particles	2707	Sub-tomogram	9.7		[67]

	HIV-1	Protease inhibitor treated	2706	Sub-tomogram	8.8	4usn	[67]
		CANC tube	2638	Helical	9.4	4d1k	[80] 11

CA Lattice	RSV	Ico. assembly of CA^2^	1862	Single particle 9	10.4		[70]
		Ico. assembly of CA/spacer 3	5772	Single particle	8.5		[32]

	HIV-1	CA A92E Tube	5582	Helical	8.6	3j34, 3j4f	[69]
		Full-length CA	1529	2D crystal. 10	9.0	3dik	[72]

Env Protein Complexes	HIV-1	gp160^4^ trimer	5418	Single particle	10.8		[81]
		gp140^5^ with 17b Fab	5462	Sub-tomogram	8.8		[60]
		gp140 trimer^6^	5779	Single particle	5.8	3j5m	[74]
		gp140 trimer^6^/2 PGV04 Fabs	5780	Single particle	7.9		[74]
		gp140 trimer^6^/3 PGV04 Fabs	5781	Single particle	8.2		[74]
		gp140 trimer^5^	2484	Single particle	6.0	4cc8	[73]
		gp160^4^ trimer	5447	Single particle	6.0		[82] 12

1. The tubular structure formed by CA and NC domains of respective virus.

2. Icosahedral assembly of RSV CA proteins.

3. T=1 Icosahedral assembly of RSV CA proteins with spacer peptide.

4. HIV-1 Env trimer precursor.

5. KNH1144 SOSIP gp140.

6. BG505 SOSIP.664.

7. Helical reconstruction.

8. Sub-tomogram averaging.

9. Single particle reconstruction.

10. Two-dimensional electron crystallography.

11. The arrangement of CTD is similar to those in the immature HIV-1 particles treated with the protease inhibitor amprenavir, while the NTD adopts a presumably non-physiological form [67].

12. The validation of the structure has been questioned and the controversy is unsettled [83–87].

## Abbreviations

AIDS: acquired immune deficiency syndrome
HIV-1: human immunodeficiency virus type 1
HTLV-1: human T-cell luekemia virus type 1
RSV: Rous sarcoma virus
MLV: murine leukemia virus
MPMV: Mason-Pfizer monkey virus
ASLV: avian sarcoma/leukosis virus
SIV: simian immunodeficiency virus
MoMLV: Moloney murine leukemia virus
FV: Foamy virus
MMTV: mouse mammary tumor virus
MA: matrix protein
CA: capsid protein
NTD: amino-terminal domain of capsid protein
CTD: carboxyl-terminal domain of capsid protein
NC: nucleocapsid protein
Env: envelope glycoprotein
MHR: major homology region
PI(4,5)P2: phosphatidylinositol 4,5-bisphosphate
TRIM5α: tripartite motif protein isoform 5 alpha
bn-MAbs: broadly neutralizing monoclonal antibodies
CD4bs: CD4 binding site
CD4i: CD4-induced
HUVEC: human umbilical vein endothelial cells
ESCRT: endosomal sorting complexes required for transport
TEM: transmission electron microscopy
cryo-EM: cryo-electron microscopy
cryo-ET: cryo-electron tomography
NMR: nuclear magnetic resonance
STEM: scanning transmission electron microscopy
FFS: fluorescence fluctuation spectroscopy
FIB-SEM: focused ion beam scanning electron microscopy
